# ZNF768 loss amplifies p53 action and reduces lung tumorigenesis in mice

**DOI:** 10.1038/s41388-025-03352-w

**Published:** 2025-03-25

**Authors:** Audrey Poirier, Timon Utecht, Romain Villot, Yves Gélinas, Mathilde Mouchiroud, Manal Kordahi, Alona Kolnohuz, Coline Pasteur, Joanny Roy, Marie-Josée Beaulieu, Michèle Orain, Nolwenn Samson, Marie-Renée Blanchet, Philippe Joubert, Mathieu Laplante

**Affiliations:** 1 Centre de recherche de l’Institut universitaire de cardiologie et de pneumologie de Québec (CRIUCPQ), Université Laval, Québec, QC Canada; 2 Centre de recherche sur le cancer de l’Université Laval, Université Laval, Québec, QC Canada; 3 Faculté de médecine, Université Laval, Québec, QC Canada

**Keywords:** Cancer, Mechanisms of disease, Senescence

## Abstract

Cell proliferation is a fundamental process required for organismal development, growth, and maintenance. Failure to control this process leads to several diseases, including cancer. Zinc finger protein 768 (ZNF768) is an emerging transcription factor that plays key roles in driving proliferation. In addition to controlling a gene network supporting cell division, ZNF768 physically interacts and inhibits the activity of the tumor suppressor p53. Although the importance of ZNF768 in promoting cell proliferation has been well demonstrated in vitro, the physiological and pathological roles of ZNF768 in vivo are still unknown. Here, we report the generation and characterization of a ZNF768 null mouse model. ZNF768 null mice are viable but show a growth defect early in life. Mouse embryonic fibroblasts (MEFs) isolated from ZNF768 null embryos exhibit higher p53 levels, premature senescence, and higher sensitivity to genotoxic stress. In line with these findings, ZNF768 null mice showed increased radiosensitivity. This effect was associated not only with higher expression of a subset of p53 target genes, but also with alterations in genes regulating transmembrane receptor signaling, cell adhesion, and growth. Because ZNF768 levels are elevated in tumors, we tested the impact of ZNF768 loss on cancer development in mice. Here, we show that ZNF768 deletion was sufficient to repress lung tumor development in a KRAS^G12D^-induced cancer mouse model. Overall, our findings establish ZNF768 as an important protein controlling cell proliferation that could potentially be targeted to reduce tumorigenesis.

## Introduction

Cell proliferation is a fundamental process required for organismal development, growth, and maintenance. Ensuring a tight control of cell division is essential to sustain both tissue and systemic homeostasis. Over the last decades, several proteins involved in growth factor signaling, cell cycle progression, and DNA repair have been identified to play crucial roles in regulating cell proliferation [1]. One of these important regulators is tumor protein 53 (p53). Often referred to as the “guardian of the genome”, p53 is activated in response to genotoxic stress to trigger the expression of genes promoting cell cycle arrest, apoptosis, and senescence and prevent replication of damaged cells [2]. Mutations that inactivate p53 and other proliferative checkpoints are playing a central role in cancer development [2, 3]. Extending the understanding of the mechanisms regulating cell proliferation is a crucial step toward the development of new strategies to fight cancer.

Zinc finger protein 768 (ZNF768) was recently identified as a novel transcription factor controlling cell fate decision and proliferation in normal and cancer cells in vitro [4, 5]. ZNF768 is a conserved protein that contains heptad-repeat structures related to the C-terminal domain (CTD) of the large subunit of RNA polymerase II (RPB1), a unique feature among its class [4, 5]. This transcription factor associates with euchromatin and promoter regions of many genes in a cell type-specific manner in human cells [5]. Specifically, it was observed in two independent studies that ZNF768 binds to the core region of Mammalian-wide interspersed repeats (MIRs), a group of retrotransposed DNA elements associated with transcriptionally active euchromatin [5, 6]. Based on these experiments, it has been proposed that ZNF768 binding is associated with cell type-specific gene expression and that this transcription factor evolved to extend the repertoire of gene regulatory mechanisms in mammals [5]. The initial characterization of ZNF768 showed that it strongly associates with proliferation in cultured cells [4, 5]. We and others have shown that ZNF768 is required for cell growth and survival and that its depletion induces a strong antiproliferative and pro-apoptotic response in several cell lines in vitro [4, 5]. Interestingly, hyperactivation of RAS and DNA damage, that both impair proliferation, severely reduce ZNF768 stability in cell lines [4]. It was reported that stress-mediated degradation of ZNF768 is associated with reduced expression of numerous pro-proliferative genes and with a rise in the levels of several targets of p53 [4]. Notably, ZNF768 overexpression was reported to be sufficient to bypass RAS-induced senescence in vitro in a p53-dependent manner [4]. Supporting a link between ZNF768 and p53, we and others have found that ZNF768 interacts with p53 [4, 6]. Furthermore, we showed that ZNF768 represses p53 phosphorylation and activation [4]. Based on these results, we proposed a model in which genotoxic stress downregulates ZNF768 levels to repress the expression of key cell cycle effectors, amplify p53 activation, and reduce cellular proliferation and survival [7].

Owing to the close relationship between ZNF768 and cell proliferation, the expression level of this transcription factor was analysed in various cancer using public databases. These analyses revealed that *ZNF768* overexpression is common in a variety of cancers in humans [4]. In-house validation studies using a lung cancer cohort confirmed that ZNF768 protein levels are elevated in tumors [4]. In a follow-up study, ZNF768 levels positively correlated with proliferative clinicopathological features of lung adenocarcinomas (LUAD) such as Ki-67 and the mitotic score [8]. Furthermore, depleting ZNF768 expression in lung cancer cell lines severely repressed proliferation [8]. Altogether, these results suggest that cancer cells might take advantage of ZNF768 to sustain tumorigenesis.

Although the importance of ZNF768 in promoting cell proliferation has been well demonstrated in vitro, the physiological and pathological roles of ZNF768 in vivo are still unknown. To study the functions of ZNF768 in vivo, we generated a ZNF768 knockout mouse model using the CRISPR/Cas9 approach. Here, we report that ZNF768 null mice are viable but show a growth defect early in life that resorbs when animals reach adulthood. Supporting a link between ZNF768 and p53 activation, we found that mouse embryonic fibroblasts (MEFs) isolated from ZNF768 null embryos exhibited higher p53 activation, premature senescence, and higher sensitivity to genotoxic stress. Surprisingly, the rise in p53 activation state observed in ZNF768 null MEFs was not apparent in the tissues of ZNF768 knockout mice in basal state. However, we found that ZNF768 loss increased radiosensitivity in mice, an effect linked to higher expression of p53 targets and altered expression of genes regulating transmembrane receptor signaling, cell adhesion, and growth. We also found that ZNF768 loss increased expression of cell cycle inhibitor p21 in a carcinogen-induced cancer mouse model and repressed lung tumor development in response to oncogenic KRAS. These results support a role of ZNF768 in the cellular stress response in vivo and suggest that ZNF768 could be targeted to reduce tumorigenesis.

## Material and methods

### Mouse models

*Znf768* knockout mice were generated by the McGill Integrated Core for Animal Modeling (MICAM) using the CRISPR-Cas9 technology. All experiments followed the guidelines of the Canadian Council of Animal Care and were approved by McGill University’s Animal Care Committee. Guide RNA (gRNA) sequences were designed using the MIT website (www.crispr.mit.edu) and synthesized using a T7 transcription kit (Thermofisher, #AM1354). Each gRNA was tested for cutting efficiency through microinjection into zygotes of C57BL/6 N embryos along with mRNA Cas9 (Sigma-Aldrich, #CAS9MRNA). These embryos were cultured until reaching the blastocyst stage (E3.5) when DNA was extracted for PCR and sequenced. PCR was performed with the following primers (5’- CTCCAGGCACTCGAACTGG-3’, 5’-GGGGTCTGGAAGGTCATTGG-3’) and then sent for sequencing. The chosen gRNA (TTCGGGGCTGCCCACTGCGT) and mRNA Cas9 were subsequently utilized for microinjection into mouse zygotes derived from C57BL/6 N mice at a concentration of 20 ng/µl each. These embryos were then implanted into pseudopregnant CD-1 females for gestation, resulting in the generation of founder mice. LSL-*KRAS*^*G12D*^ and LSL-*KRAS*^*G12D*^*; Trp53*^*Flox*^ mice were obtained from The Jackson Laboratory (strain #008179 and # 032435 respectively) and have been described previously [9, 10]. LSL-*KRAS*^*G12D*^*; Znf768*^*−/−*^ were generated by crossing the two single mutant strains LSL-*KRAS*^*G12D*^ (JAX, strain #008179) and *Znf768*^*−/−*^ mice.

### Genotyping

Genotyping of *Znf768* mice was performed with the following primers: *Znf768* forward = 5’—CTCCAGGCACTCGAACTGG—3’, *Znf768* reverse = 5’—GGGGTCTGGAAGGTCATTGG—3’. Using these primers, an amplicon of 402 bp is produced corresponding to the wild-type *Znf768* allele. A fragment of 385 bp is produced corresponding to the *Znf768* knockout allele. The PCR products were run on a 3% agarose gel for 3 h to separate and detect the amplicons.

### Isolation and culture of primary MEFs

Mouse embryonic fibroblasts were derived from E13.5 embryos from *Znf768* heterozygous breeding pairs. After removing the head, liver, and heart, the embryos were minced and trypsinized for 10 min and then seeded into TC-150 cell culture dishes. The cell lines were cultured in complete Dulbecco’s Modified Eagle Medium (DMEM) (Wisent, #319-005-CL) supplemented with Fetal Bovine Serum (FBS) (10%) (Sigma-Aldrich, #F1051) and penicillin- Streptomycin (1%) (Wisent, #450-201-EL). The cell lines were cultured according to standard mammalian culture protocols and sterile techniques. The primary MEFs were not tested for mycoplasma contamination. DNA damage was induced in MEFs using Doxorubicin (Tocris, #2252). For clonogenic assays, primary MEF cells were plated at low confluency and subjected to irradiation 24 h after plating. Cells were incubated for 14 days after which colonies were fixed 20 min in methanol (Fisher Scientific, #A412-4) and stained 40 min with a crystal violet solution (0.5% w/v crystal violet Sigma Aldrich # C6158-50G, 25% methanol). Plates were washed with dH20 and allowed to dry overnight. Absorbance at 595 nm was measured following dissolution of the dye with a 10% acetic acid solution (VWR, #CA71006-424). Absorbance was reported on the non-irradiated control for both wild-type and knockout cell lines. Blinding was not performed for the all the in vitro experiments.

### Animal experiments

All experimental protocols were approved by the Animal Ethics Committee of Université Laval (CPAUL) and were performed following the guidelines of the Canadian Council on Animal Care. The reference numbers of the approved protocols are 2019-244, 2020-601, and 2022-1022. All mice were maintained on a 12:12 h light–dark cycle, while housed in ventilated cages at ambient temperature (23 ± 1 °C). The animals were fed a normal chow diet. For the irradiation studies, 3-month-old mice were subjected to 4 Gy total body irradiation at a dose rate of 1 Gy/min (X-RAD 225XL irradiator; Precision X-Ray, Madison, CT, USA). Following the irradiation, mice were housed in sterile conditions and monitored for signs of irradiation sickness. For tumor initiation with 3-methylcholanthrene (3-MC) (Sigma-Aldrich, #213942), 5 months old mice of both sexes received a single intramuscular injection of 1 µg 3-MC dissolved in 50 µl vegetable oil in one of the rear legs. Animals were monitored twice per week after onset of tumor growth was observed in the first animal. Subcutaneous tumor volume was quantified by measuring the diameter of tumors using a digital caliper. Tumor size was not allowed to exceed 1.5 cm in diameter in accordance with institutional guidelines. For lung tumor initiation, 7–9 weeks old LSL-*KRAS*^*G12D*^, LSL-*KRAS*^*G12D*^*; Trp53*^*Flox*^, and LSL-*KRAS*^*G12D*^*; Znf768*^*−/−*^ mice were injected intratracheally with 2.5 * 10^7^ PFU of pAd5CMVCre-mCherry (Viral Core Facility, University of Iowa, USA, #649). Animals were monitored once per week after infection until sacrifice. Mice were randomly assigned to their experimental group without exclusion. Blinding was not done during the animal experiments.

### RNA extraction, sequencing, and data processing

Total RNA was isolated from tissue using TRI Reagent Solution (ThermoFisher Scientific, #AM9738) and the Monarch Total RNA Miniprep Kit (NEB, #T2010) and RNA concentration was estimated from absorbance at 260 nm. The NEBNext Ultra II directional RNA library prep kit for Illumina (NEB, #E7760L) was used to prepare mRNA sequencing libraries using 800 ng of total RNA as a template. Adapter ligated DNA was purified with the AxyPrep Mag PCR Clean-up kit (Axygen, #MAG-PCR-CL) and a PCR enrichment step of 9 cycles was done. The quality of final amplified libraries was examined with a DNA screentape D1000 on a TapeStation 2200 and the quantification was done on the QuBit 3.0 fluorometer (ThermoFisher Scientific). Subsequently, mRNA-seq libraries with unique dual index were pooled together in equimolar ratio and sequenced for paired-end 100 pb sequencing on an Illumina NovaSeq 6000 at the Next-Generation Sequencing Platform, Genomics Center, CHU de Québec-Université Laval Research Center, Québec City, Canada. The mean coverage/sample was 35 M paired-end reads. The quality of sequencing reads was first assessed using FastQC (version 0.12.0) [11]. Adapter sequences and low-quality bases were removed using Trim Galore (version 0.6.10) [12] and the reads were next aligned to the mouse genome (GRCm39) using Kallistoworkflow (version 0.46.1) [13]. Tximport R package (version 1.34.0) [14] was applied to import transcript abundances and obtain estimated counts. Differential gene expression analysis was performed with theDESeq2 R package (version 1.46.0) [15], and genes with an adjusted *p* < 0.1 were considered differentially expressed. For heatmap visualization, TPM data was log2-transformed, and fold changes were calculated. Gene enrichment analysis was performed using Metascape [16].

### Gene and protein expression analysis

Western blotting and quantitative real-time PCR were performed as described previously [4]. For western blotting, the following primary antibodies were used: ZNF768 (Aviva Systems Biology, FLJ23436, dilution 1 :1000), β-actin [Cell Signaling Technology, #4967, dilution 1:1000], Phospho-histone H2A.X (Ser139) [20E3, Cell Signaling Technology, #9718, dilution 1:1000], p53 [1C12, Cell Signaling Technology, #2524, dilution 1:1000], p21 Waf1/Cip1 [Cell Signaling Technology, #64016, dilution 1:1000], GAPDH [1D4, Novus Biologicals, dilution, NB300-221 1:1000]. Secondary antibodies were purchased for Cell Signaling Technology [Cell Signaling Technology, #7074, #7075, dilution 1:5000]. For quantitative real-time PCR, the following primers were used (forward, reverse): *Cdkn1a* (GACAAGAGGCCCAGTACTTCC, CTTGCAGAAGACCAATCTGCG), *p16ink4a* (TGTGCATGACGTGCGGG, TAGTGGGGTCCTCGCAGTT), *Il6* (GTTCCTCTCTGCAAGAGACTTCCA, CACGATTTCCCAGAGAACATGTGT), *Mmp3* (CTCGTGGTACCCACCAAGTC, CTCGTGGTACCCACCAAGTC), *Ccl2* (CCCAATGAGTAGGCTGGAGA, TCTGGACCCATTCCTTCTTG), *Ccna2* (GATGGCAGTTTTGAATCACCACA, CTCAACCAGCCAGTCCACAA), *Ccnb2* (CAAAGTACCAGCTCTGCCCA, ACTGTAAAACCTCAAGCTGCCT), *Mki67* (CCAGCACTCCAAAGAAACCC, TGCTGCTTCTCCTTCACTGG), *Pcna* (GCAGATGTGCCCCTTGTTGTA, AGAAAAGACCTCAGGACACGC), *Mcm2* (CTCCAAGGCTGGCATCGTTA, GCCAGCATCTCATCCTGAAC), *Gadd45a* (AGGGACTCGCACTTGCAATA, GGGTCTACGTTGAGCAGCTT), *Serpine1* (GGGCTCGAGTATGACGTTGT, GAGCTGCTCTTGGTCGGAA), *Pml* (AGAGGATTCGGACACCGAGA, TGGCTAATTTTCTGGGTTTCATTG), *Bax* (AGGATGCGTCCACCAAGAAG, CTTGGATCCAGACAAGCAGC), *Fas* (TGCAGACATGCTGTGGATCTG, GGCATGGTTGACAGCAAAATG), *Bbc3* (TGAGACGCGGCATAGAGCC, GGGCTAGACCCTCTACGGG), *Znf768* (GTAGGATCGTCCGCCATCG, TTCTCACTTGTGTTGCCTGC), *Mdm2* (TCAGGATCTTGACGATGGCG, AAGCCAGTTCTCACGAAGGG), *Actb* (CTCTAGACTTCGAGCAGGAG, AGAGTACTTGCGCTCAGGAG), *Arbp* (AGAAACTGCTGCCTCACATC, CATCACTCAGAATTTCAATGG), *B2m* (GACCGGCCTGTATGCTATCC, CATGCTTAACTCTGCAGGCG), *Gapdh* (GGCAAATTCAACGGCACAGT, CTCGTGGTTCACACCCATCA), *Hprt* (GGTTAAGCAGTACAGCCCCA, TGCAGATTCAACTTGCGCTC), *18* *s* (GGCCTGCGGCTTAATTTGAC, CATGCCAGAGTCTCGTTCGT).

### Tissue staining and immunohistochemistry

For immunohistochemistry (IHC), samples were fixed 24 h in 10% buffered formalin phosphate (Thermofisher, #SF100) and processed for paraffin embedding. Five-micrometer-thick sections were cut from formalin-fixed paraffin-embedded tissue sections on a microtome and placed on charged slides. Staining with ZNF768 antibody was performed as described previously [8].

### Slide digitalization and tumor scoring

Tumor analysis was done on digitalized slides. Whole slide images of each tissue slides stained with standard H&E and all IHCs were obtained at 20X magnification using a slide scanner (NanoZoomer 2.0-HT; Hamamatsu, Bridgewater, NJ, USA). Whole slide images were visualized using the companion software (NDP view, Hamamatsu, Japan). All slides were reviewed by a pathologist for analysis of tumor burden and histological grade of the lesions. Tumor burden was manually calculated as the tumor area over total lung area and was assessed on 2 separate lung sections for each mouse. Classification of the lung lesions has been described previously [9]. Evaluation of the tumor burden and histological grade were performed in a blinded manner. Scoring of protein expression was done using software for bioimage analysis (QuPath; Queen’s University, Belfast, Northern Ireland). ZNF768 expression was evaluated using the H-score method. This method combines the percentage of stained nuclei and the staining intensity using the following equation to obtain a score between 0 and 300: % nuclei of intensity 1 × 1 + % nuclei of intensity 2 × 2 + % nuclei of intensity 3 × 3.

### Statistics and reproducibility

The appropriate statistical tests were determined based on the research objective and the nature of the variables. All appropriate tests were done to ensure the data meets the assumptions of the statistical tests used. Statistical analyses were performed using Prism (version 9.0.2) and R (version 4.4.2). The statistical tests used to determine significance, and the sample size are identified in the figure legends. Sample size was estimated based on previous experience with the experimental approaches. Key findings in cells were reproduced in at least 2 independent experiments or in different cell lines. Animal studies were performed once using several animals per groups to insure significance.

## Results

### Generation of a ZNF768 knockout mouse model

**Fig. 1:**
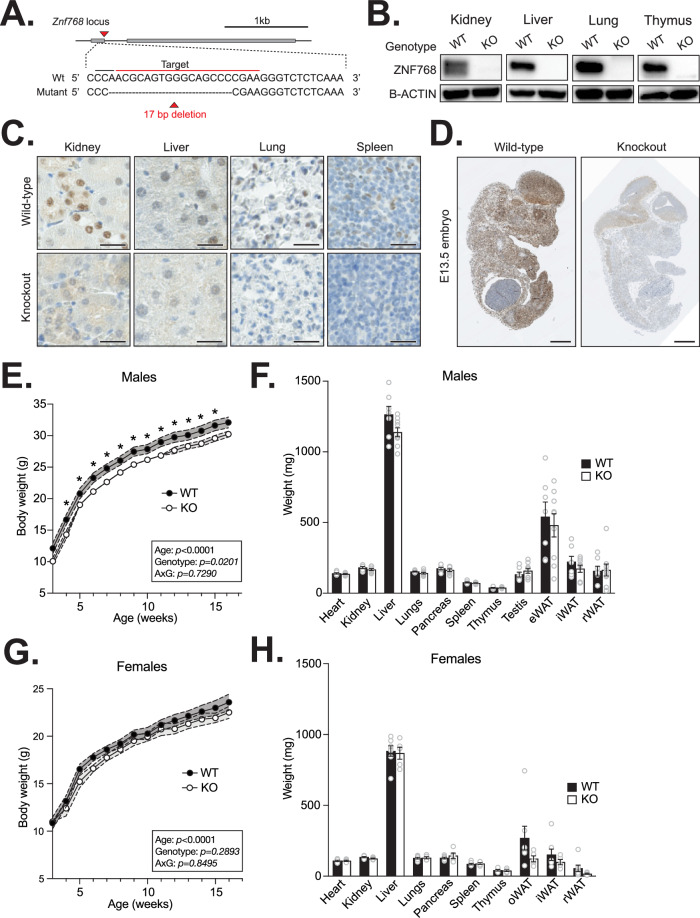
Generation of a ZNF7678 knockout mouse model. **A** Schematic overview of the *Znf768* knockout allele generated by CRISPR-Cas9. The sgRNA targets the first exon of *Znf768* (gray boxes). Mutant *Znf768* allele contains a 17 bp deletion causing a frame shift in *Znf768* sequence and the premature disruption of its coding sequence. **B** Western blot showing the expression of ZNF768 in tissues of ZNF768 wild-type and knockout mice. B-ACTIN was used as a loading control. **C** ZNF768 immunohistochemistry on tissues of ZNF768 wild-type and knockout mice (magnification ×40, scale bars 25 µm). **D** ZNF768 immunohistochemistry on whole ZNF768 wild-type and knockout E13.5 embryos (magnification ×1.25, scale bars 1 mm). Body weight (*n* = 12-18 animals/group) and tissue weight (*n* = 5–9 animals/group) of ZNF768 wild-type and knockout (**E**, **F**) male and (**G**, **H**) female mice respectively. Mice were sacrificed at 21-22 weeks of age. Data represent the mean ± SEM. For body weight, significance was determined by mixed model analysis *P < 0.05 versus controls. For tissue weight, significance was determined by 2-tailed, unpaired *t*-test. *P < 0.05 versus controls.

To investigate the physiological role of ZNF768, we have generated a complete knockout mouse model using the CRISPR-Cas9 technology. Using a single guide RNA (sgRNA) targeting the first exon of *Znf768*, we produced a mutated *Znf768* allele with a 17 base pair deletion causing a frameshift in *Znf768* sequence and the premature disruption of its coding sequence (Figs. 1A and S1A). Loss of ZNF768 expression was confirmed in various tissues by western blot and immunohistochemistry (Fig. 1B–D). Based on our previous work in vitro [4], we expected severe developmental defects in response to ZNF768 deletion. Unexpectedly, ZNF768 null mice were born at the expected Mendelian ratio, were viable, and did not display any gross abnormalities (Figs. S1B and 1D). As presented in Fig. 1E, we observed that male ZNF768 knockout mice were slightly smaller compared to wild-type animals until 15 weeks of age, suggesting a mild growth defect. However, no differences in body weight, tissue weight, and length were observed at a later time point, indicating that ZNF768 null mice eventually catch up to wild-type animals in adulthood (Figs. 1F and S1C). Female knockouts also showed a tendency for a lower body weight, but this effect was not significant (Figs. 1G and S1D). No difference in tissue weight was observed in response to ZNF768 loss in females (Fig. 1H). Altogether, these results indicate that ZNF768 is not an essential gene and that its depletion causes a small growth defect but does not prevent overall development in mice.

### Loss of ZNF768 increases p53 levels and causes premature senescence in primary MEFs

**Fig. 2:**
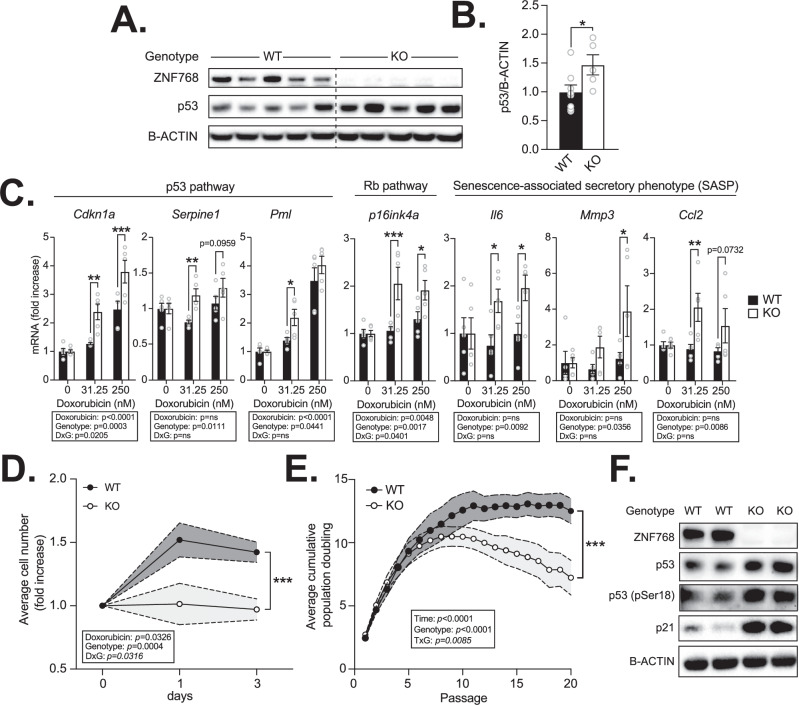
ZNF768 depletion increases p53 levels and causes premature senescence in MEFs. **A** Western blot showing the expression of ZNF768 and p53 in ZNF768 wild-type and knockout MEFs at P4. B-ACTIN was used as a loading control. Representative samples are shown. **B** Quantification of p53 protein levels from the Western blot described in (**A**) (*n* = 5-8 cell lines/group). Data represent the mean ± SEM. Significance was determined by 2-tailed, unpaired *t*-test. *P < 0.05 versus controls. **C** ZNF768 wild-type and knockout MEFs were treated with doxorubicin for 24 h (0 to250 nM). The cells were next washed and followed for 48 h and RNA was harvested. The expression of indicated genes was measured by RT-qPCR (*n* = 5 cell lines/group). Data represent the mean ± SEM. Significance was determined by 2-way ANOVA. *P < 0.05, **P < 0.01 ***P < 0.001 versus controls. **D** ZNF768 wild-type and knockout MEFs were treated as described in (**C**) and counted starting at doxorubicin treatment (*n* = 5-8 cell lines/group). Data represent the mean ± SEM. Significance was determined by 2-way ANOVA. ***P < 0.001 versus controls. **E** Growth curve of ZNF768 wild-type and knockout MEFs cultured under the standard 3T3 protocol (*n* = 4 cell lines/group). Data represent the mean ± SEM. Significance was determined by 2-way ANOVA. ***P < 0.001 versus controls. This experiment was reproduced twice in distinct cell lines. **F** Western blot showing the expression of ZNF768, total and phosphorylated p53, and p21 in ZNF768 wild-type and knockout MEFs at P7. B-ACTIN was used as a loading control. Representative samples are shown.

We have previously shown that ZNF768 depletion greatly impairs cell proliferation and survival in vitro, an effect associated with decreased expression of key genes regulating cell cycle and the rise in the expression of numerous p53 target genes [4]. In this report, we showed that ZNF768 interacts with p53 to repress its activation. To define whether ZNF768 impacts p53 in our model, we first measured the effect of ZNF768 loss on p53 levels in mouse embryonic fibroblasts (MEFs). Here, we observed significantly higher levels of p53 protein in ZNF768 null MEFs suggesting higher basal activation of p53 (Fig. 2A and B). We next tested the acute regulation of p53 activity in MEFs using the DNA damaging agent doxorubicin. Precisely, MEFs were treated with low doses of doxorubicin for one day, after which they were washed and allowed to rest for 2 days. This treatment was previously reported to activate p53 and to induce senescence in various cell types [4, 17–20]. Supporting higher p53 activity in the absence of ZNF768, we observed a significant rise in the expression of the classical p53 target genes *cyclin dependant kinase inhibitor 1* *A* (*Cdkn1a*, also known as *p21*), *PML nuclear body scaffold (PML)*, and *serpin family E member 1* (*Serpine1*, also known as *Pai1*) in ZNF768 null MEFs exposed to doxorubicin (Fig. 2C). In line with these findings, ZNF768 null cells rapidly stopped proliferating and displayed increased expression of multiple senescence markers following doxorubicin treatment (Fig. 2C and D). We next tested the impact of ZNF768 loss on replicative crisis in MEFs using the 3T3 method. This protocol is commonly used to study cellular transformation and tumorigenesis in primary cells, two processes tightly linked to p53 activation [21, 22]. As presented in Fig. 2E, a reduction in proliferation was rapidly observed in ZNF768 knockout MEFs. In this experiment, we found that ZNF768 null cells prematurely entered replicative crisis following serial passage, a phenotype reminiscent of elevated p53 activation (Fig. 2E). To confirm the impact on p53, we looked at p53 total levels and its phosphorylation state, two parameters recognized to play key roles in p53 function [23]. Supporting an elevated p53 activation in ZNF768 null cells, we observed an increase in the total levels and phosphorylation of p53 after several passages (Fig. 2F). Overall, these results show that ZNF768 loss increases p53 levels and impairs proliferation in response to DNA damage and replicative stress.

### ZNF768 loss does not hyperactivate p53 in mouse tissues in the basal state

Based on the observations made in vitro, we next tested whether the loss of ZNF768 was sufficient to activate p53 and elicit an antiproliferative, and possibly pro-apoptotic response, in tissues of ZNF768 null mice. Precisely, we measured the expression of proliferative markers and genes coding for proteins regulating cell cycle arrest, cellular senescence, and cell death in thymus, heart, and liver of male and female wild-type and ZNF768 null mice. Contrary to our in vitro observations, we found that ZNF768 loss did not cause a significant shift in the expression of these genes, including multiple p53 targets, in any of the tissue tested, in both males and females (Fig. S2A–C).

### Identification of physiological conditions regulating ZNF768 levels in vivo

**Fig. 3:**
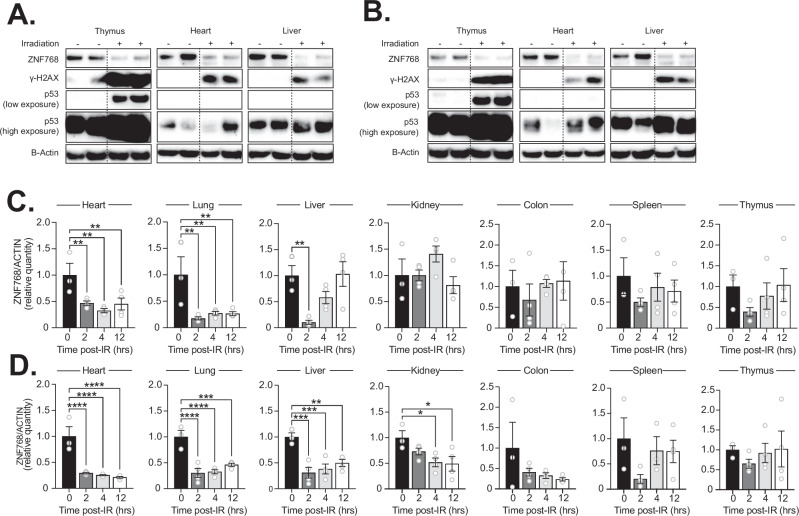
ZNF768 levels are decreased following total body irradiation in mice. Western blot analysis of ZNF768, γ-H2AX, and p53 in tissues of (**A**) male and (**B**) female wild-type mice 2 h after 4 Gy total body irradiation. B-ACTIN was used as a loading control. Protein quantification by Western blot of ZNF768 in tissues of (**C**) male and (**D**) female wild-type mice 0, 2, 4, and 12 h after 4 Gy total body irradiation (*n* = 3-4 animals/group). B-ACTIN was used as a loading control. Data represent the mean ± SEM. Significance was determined by 1-way ANOVA. *P < 0.05, **P < 0.01 ***P < 0.001 ****P < 0.0001 versus controls.

The results presented above indicate that, although ZNF768 loss impacts p53 activation in MEFs, its depletion minimally affects p53 in mouse tissues in basal conditions. Based on these observations, we reasoned that exposing mice to stressful conditions modulating endogenous ZNF768 levels might be required to amplify the responses linked to its deletion in vivo. To test this possibility, we first sought to identify conditions modulating ZNF768 levels in mouse tissues. We have previously shown that ZNF768 levels are decreased upon treatment with DNA-damaging agents in vitro [4]. To define whether ZNF768 is regulated by DNA damage in vivo, ZNF768 levels were measured following total-body irradiation, a treatment that induces DNA damage, p53 activation, senescence, and cell death in multiple organs and tissues [24–30]. Here, wild-type mice were exposed to a single total body irradiation of 4 Gray (Gy) and were sacrificed 2 h post-irradiation. As expected, we observed an increase in DNA damage markers and the accumulation of p53 following irradiation (Fig. 3A and B). Supporting the connection between ZNF768 and DNA damage, a significant decrease in ZNF768 protein levels was observed in the thymus, heart, and liver following irradiation in both male and female mice (Fig. 3A and B). To extend these observations, additional tissues were collected from mice at 2, 4, and 12 h post-irradiation. The decrease in ZNF768 protein levels remained significant in most tissues and was particularly severe in the heart, lungs, and liver (Fig. 3C and D). Interestingly, this effect was not associated with any decrease in *Znf768* mRNA expression, indicating that irradiation impacts ZNF768 post-transcriptionally (Fig. S3A and S3B).

**Fig. 4:**
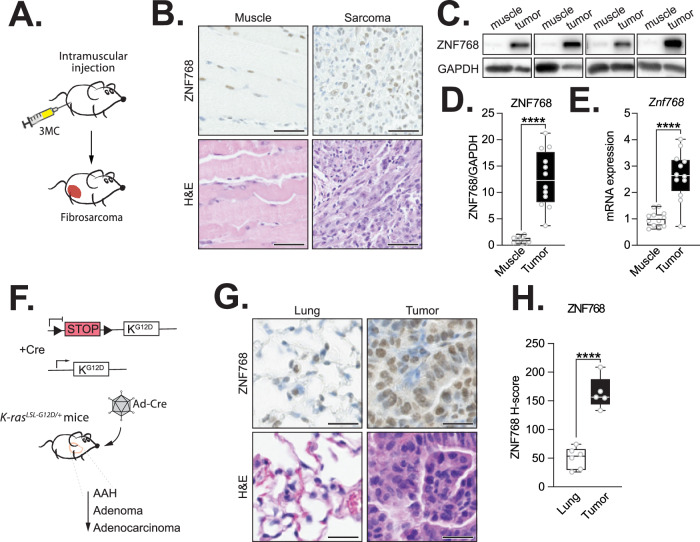
ZNF768 levels are induced in chemical and genetically engineered cancer mouse models. **A** Schematic representation of the strategy used to induce sarcoma in mice with 3-methylcholanthrene (3MC). Following 3MC injection in the hindlimb, mice developed high-grade sarcoma with short latency (tumors in red). **B** ZNF768 immunohistochemistry and H&E staining on muscle and 3MC-induced sarcoma of wild-type mice (magnification ×20, scale bars 50 µm). **C** Western blot of ZNF768 protein level in muscle and 3MC-induced sarcoma of wild-type mice. GAPDH was used as a loading control. **D** Quantification of ZNF768 protein levels from the Western blot described in (**C**) (*n* = 12 animals/group). Data are presented as box plots, where the center line represents the median, the box extends from the first to the third quartile and the whiskers indicate the minimum and maximum values. Significance was determined by 2-tailed, unpaired t-test. ****P < 0.0001 versus controls. **E** *Znf768* transcript expression in muscle and 3MC-induced sarcoma of wild-type mice (*n* = 5-8 animals/group). Data are presented as box plots. Significance was determined by 2-tailed, unpaired *t* test. ****P < 0.0001 versus controls. **F** Schematic representation of the genetic strategy used to induce lung adenocarcinoma. The LSL-*KRAS*^*G12D*^ mice carry the conditional activatable LSL-*KRAS*^G12D^ allele. The silenced LSL allele comprises the *KRAS* gene carrying a point mutation (G12D) whose expression is blocked by the presence of a loxP-flanked stop codon. Following the intratracheal injection of Ad-Cre virus, the Cre-mediated recombination allows the expression of oncogenic *KRAS*^*G12D*^ and the development of lung tumor lesions ranging from atypical adenomatous hyperplasia (AAH) to adenocarcinoma. **G** ZNF768 immunohistochemistry and H&E staining on normal lung and lung tumors (magnification ×40, scale bars 25 µm). **H** Quantification of ZNF768 immunochemistry staining using the H-score in lung and lung tumors (*n* = 5-6 tumors/group). Data are presented as box plots. Significance was determined by 2-tailed, unpaired *t* test. ****P < 0.0001 versus controls.

We have previously shown that *ZNF768* is amplified and/or overexpressed in various cancer types in humans [4, 8]. In lung cancer, we found that ZNF768 protein levels positively correlate with Ki-67 and other proliferative clinicopathological features [8]. To define whether ZNF768 levels are similarly regulated in mice, we measured ZNF768 levels in two established models of cancer. We first generated fibrosarcoma by injecting the carcinogen 3-methylcholanthrene (3MC) in the hind limb of wild-type mice (Fig. 4A). As shown before, 3MC generates high-grade tumors as confirmed histologically (Fig. 4B) [31]. Here, we observed that ZNF768 levels were highly increased in fibrosarcoma compared to normal muscle (Fig. 4B–E). In these experiments, we found that both ZNF768 protein and mRNA levels were increased in tumors. In a second mouse model, we tested the regulation of ZNF768 in lung tumors induced by oncogenic RAS activation. Briefly, mice carrying a *Lox-Stop-Lox* (LSL) termination sequence with the *KRAS*^*G12D*^ mutation (LSL-*KRAS*^*G12D*^) were intratracheally injected with adenovirus expressing Cre-recombinase (Ad-Cre). As shown before, this treatment promotes the recombination of the LSL cassette, the overexpression of oncogenic KRAS^G12D^, and the development of lung tumors in mice (Fig. 4F) [9]. Here, ZNF768 levels in the tumors were assessed by immunohistochemical (IHC) staining of the lungs and quantified using the H-score, as previously described [8]. As expected, lung tumors showed elevated levels of ZNF768 protein expression compared to normal lung tissue (Fig. 4G and H). These findings were also reproduced in higher-grade lung tumors induced by oncogenic RAS activation and p53 loss. In this model, mice carry Lox sites flanking the *p53* gene in addition to the LSL-*KRAS*^*G12D*^ cassette (LSL-*KRAS*^*G12D*^*; Trp53*^*Flox*^). Following intratracheal injection of Ad-Cre, the recombination of the Lox sites induces the deletion of p53, the overexpression of oncogenic KRAS^G12D^, and the development of high-grade lung tumors (Fig. S4A) [10]. Once more, we observed an increase in ZNF768 protein levels in the lung tumors of these mice (Fig. S4B). Interestingly, ZNF768 levels were positively associated with tumor grade in this model of lung cancer (Fig. S4C). Altogether, these results show that overexpression of ZNF768 is common in various types of cancers in mice.

### Loss of ZNF768 increases radiosensitivity and alters the transcriptional response to irradiation

**Fig. 5:**
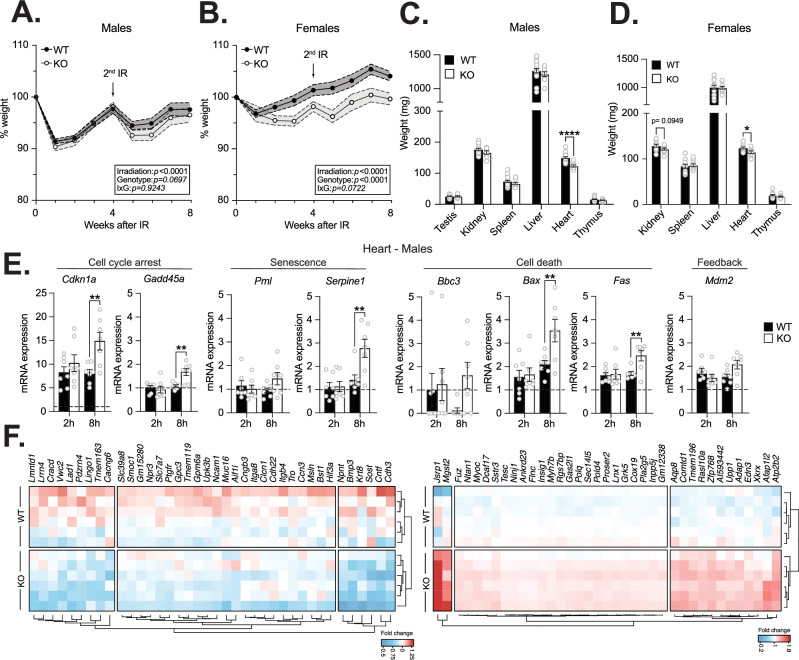
Loss of ZNF768 increases radiosensitivity and alters the transcriptional response to irradiation. Weight variations in wild-type and ZNF768 knockout (**A**) male and (**B**) female mice after total body irradiation. Mice were subjected to 2 rounds of 4 Gy total body irradiation (arrow) and weight was measured (*n* = 9-20 animals/group). Data represent the mean ± SEM. Significance was determined by 2-way ANOVA. Tissue weight of wild-type and ZNF768 knockout (**C**) male and (**D**) female mice. Mice described in (**A**, **B**) were sacrificed 8 weeks after the first round of total body irradiation, and tissue weight was measured (*n* = 9-20 animals/group). Data represent the mean ± SEM. Significance was determined by 2-tailed, unpaired *t*-test. *P < 0.05, ****P < 0.0001 versus controls. **E** qPCR analysis of p53 target genes in the heart of male wild-type and ZNF768 knockout mice 2 and 8 hours after 4 Gy total body irradiation (*n* = 7 animals/group). Data represent the mean ± SEM. Significance was determined by 2-way ANOVA. **P < 0.01 ***P < 0.001 ****P < 0.0001 versus controls. **F** Heat-map visualization of differentially expressed genes in the heart of male wild-type and ZNF768 knockout mice 8 h after 4 Gy total body irradiation (Padj < 0.01 and fold change <0.7 and >1.25; *n* = 6 animals/group). Significance was determined by Wald test with the Benjamini-Hochberg adjustment method.

As shown above, we observed that irradiation was linked to dynamic changes in ZNF768 endogenous levels in multiple tissues. Based on these observations, we hypothesized that the effect of ZNF768 loss could be amplified in this context and could result in transcriptional changes and altered p53 activation and signaling. Previous animal studies have shown that enhanced p53 activity increases radiosensitivity in different mouse models [31–33]. To test whether ZNF768 loss alters the response to irradiation, wild-type, and ZNF768 null mice were subjected to 2 rounds of 4 Gy total body irradiation and monitored for signs of irradiation sickness such as weight loss and lethargy. As presented in Fig. 5A and B, ZNF768 null mice showed signs of increased radiosensitivity following irradiation. Indeed, we observed that ZNF768 knockout lost more weight after irradiation compared to wild-type mice, an effect that was more pronounced in female mice (Fig. 5A and B). We also observed that the heart was significantly smaller in ZNF768 null animals of both sexes after irradiation (Fig. 5C and D). Supporting that ZNF768 loss increases radiosensitivity, we found that ZNF768 null MEFs had decreased ability to form colonies following irradiation using clonogenic assays (Fig. S5A).

To determine whether the observed phenotypes could be linked to higher p53 activity in ZNF768 null mice, we explored the effect of ZNF768 loss on the expression of p53 target genes in different tissues following acute irradiation. Multiple studies have shown that p53 is activated early following irradiation in tissues [24, 25]. Here, ZNF768 wild-type and knockout mice were exposed to a single dose of irradiation (4 Gy), and the animals were sacrificed 2 and 8 hours after irradiation. As expected, irradiation was linked to a rapid increase in γ-H2AX levels in mouse tissues, an effect that was not different between wild-type and ZNF768 null mice (Fig. S5B and S5C). Interestingly, we observed higher expression of established p53 target genes (ex. *Cdkn1a*, *Gadd45a*, *Serpine1*, and *Bax*) in the tissues of male ZNF768 knockout mice (Fig. 5E and S5D). Female knockouts showed a significant rise in *Cdkn1a* levels in the heart (Fig. S5E and S5F).

Profound changes in gene expression are rapidly observed after irradiation and play key roles in DNA repair, cell cycle arrest, growth control, and cell signaling [34–36]. To gain further insights on the role of ZNF768 as a transcription factor in relation to genotoxic stress in vivo, we aimed to characterize the general transcriptional impact associated with ZNF768 loss in mouse tissue in response to irradiation. To address this question, RNA-sequencing (RNAseq) was performed on the heart of wild-type and knockout male mice following acute irradiation. The heart was chosen as this tissue was found to be particularly sensitive to irradiation in ZNF768 null mice. Confirming previous results, elevated expression of established p53 target genes (*Cdkn1a*, *Gadd45a*, and *Serpine1*) was measured in the heart of ZNF768 null mice (Fig. S5G). Interestingly, we also observed that ZNF768 loss was associated with higher expression of a subset of genes previously identified to be part of the p53 network (Fig. S5G) [37]. Importantly, in addition to confirming the relation between ZNF768 loss on p53, this analysis revealed that the absence of ZNF768 also altered the expression of multiple genes outside of the p53 pathway. In detail, 607 genes were differentially expressed between wild-type and knockout mice, with 306 upregulated and 301 downregulated genes (Fig. 5F and Supplementary Table 1). Comprehensive analysis of the most downregulated genes (Padj<0.01 and fold<0.7) using Metascape revealed significant enrichment for genes linked to transmembrane receptor signaling, cell adhesion, and growth (Fig. S5H). Interestingly, we also observed that *Znf768* was one of the most upregulated genes in the ZNF768 null hearts, suggesting a possible attempt by the cells to compensate for its deletion. Taken together, these results show that ZNF768 loss increases radiosensitivity and alters the acute transcriptional response to irradiation.

### Impact of ZNF768 loss on the development of cancer in mice

**Fig. 6:**
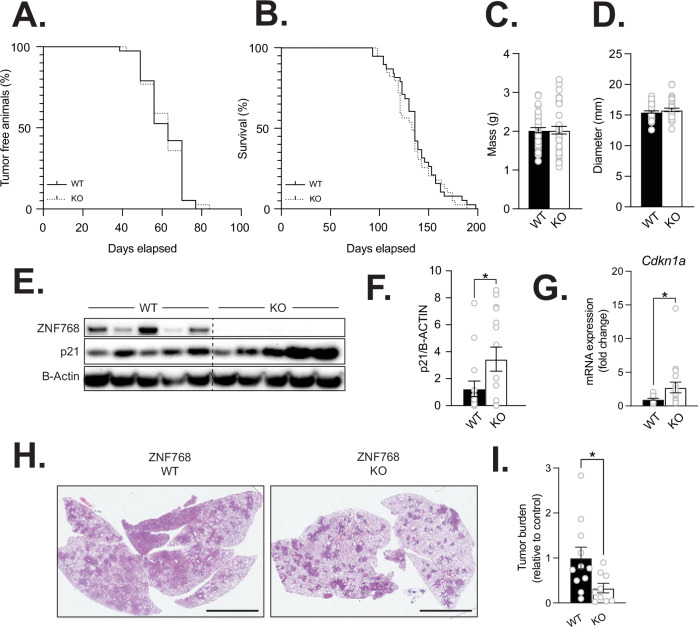
Impact of ZNF768 loss on the development of cancer in mice. Kaplan-Meier plot of (**A**) tumor incidence and (**B**) endpoint of 3MC chemical carcinogenesis of wild-type and ZNF768 knockout mice of both sexes (*n* = 38-39 animals/group). Significance was determined by log-rank test. **C** Mass and (**D**) diameter of wild-type and ZNF768 knockout 3MC sarcoma at sacrifice (*n* = 38–39 animals/group). Significance was determined by 2-tailed, unpaired *t*-test. p21/*Cdkn1a* (**E**, **F**) protein and (**G**) transcript expression in wild-type and ZNF768 knockout tumors (*n* = 15 animals/group). Significance was determined by 2-tailed, unpaired *t*-test. *P < 0.05 versus controls. **H** Representative H&E staining of lung sections from wild-type and ZNF768 knockout mice 24 weeks after KRAS lung cancer induction (magnification ×0.4, scale bars 5 mm). **I** Quantification of tumor in lung sections of wild-type and ZNF768 knockout mice described in (**H**). Significance was determined by 2-tailed, unpaired *t*-test. *P < 0.05 versus controls.

ZNF768 levels are tightly linked to proliferation in cells and tumors, where high levels of ZNF768 are commonly observed [4, 8]. We have shown that this is also the case in cancer mouse models where ZNF768 is overexpressed in tumors and associated with tumor grade. These observations suggest that ZNF768 could play an active role in tumorigenesis. To test this hypothesis, we measured the impact of ZNF768 loss on tumor development in mice. Using the 3MC fibrosarcoma model described above (Fig. 4A and B), we induced tumors in ZNF768 wild-type and knockout mice and followed these mice over approximately 6 months. Based on the impact of ZNF768 loss on proliferation and p53 activation, we expected ZNF768 loss to repress tumor development in this context. As depicted in Fig. 6A and B, we found no difference in tumor initiation and overall survival between control and ZNF768 null mice. The mass and the size of the tumors at sacrifice did not differ between the genotypes (Fig. 6C and D). Although ZNF768 loss did not induce gross changes in tumor morphology, we observed that ZNF768 null tumors had increased levels of CDKN1A (p21) (Fig. 6E–G). Such increase in the expression of p21 was not sufficient to repress tumorigenesis in the 3MC cancer model, an effect that might be attributed to the highly carcinogenic nature of the 3MC compound.

We next tested the impact of ZNF768 loss on tumor development in the oncogenic RAS-induced lung cancer model described above. In this experiment, we generated LSL-*KRAS*^*G12D*^ mice in a ZNF768 wild-type (LSL-*KRAS*^*G12D*^*; Znf768*^*+/+*^) and knockout (LSL-*KRAS*^*G12D*^*; Znf768*^*−/−*^) background. Mice were intratracheally injected with Ad-Cre virus and sacrificed 24 weeks post-infection. Confirming our initial hypothesis, we observed that ZNF768 loss repressed lung tumor development in this model. As presented in Fig. 6H and I, the overall tumor burden of the lungs was significantly decreased in ZNF768 null mice following cancer induction. In line with these findings, we found a tendency for ZNF768 knockout mice to have less advanced lung tumors. We found that the predominant lesion observed in the knockout mice was adenoma with only one animal having LUAD as the predominant lesion corresponding to 11% of the knockout mice. In comparison, 36% of the wild-type mice had LUAD as the predominant type of lesion showing more advanced disease progression. Overall, these results indicate that ZNF768 loss reduces lung tumor development induced by oncogenic KRAS^G12D^.

## Discussion

ZNF768 is an emerging regulator of cell proliferation whose expression is elevated in human cancer [4, 8]. Although the importance of ZNF768 in supporting cell proliferation and survival has been well demonstrated in cancer cells in vitro, the physiological and pathological roles of ZNF768 in vivo are still unknown. Here, we report the generation and characterization of a ZNF768 null mouse model. We show that ZNF768 null mice are viable but show a slight growth defect early in life. Supporting observations made in cancer cell lines, primary cells isolated from ZNF768 null animals exhibit higher p53 levels, premature senescence, and higher sensitivity to genotoxic stress. In mice, ZNF768 loss increased radiosensitivity and altered the acute transcriptional response to irradiation, including a subset of genes part of the p53 network. We also show that ZNF768 deletion repressed tumor development in a KRAS^G12D^-induced cancer mouse model. Overall, these findings provide physiological evidence supporting the functional relationship between genotoxic stress, ZNF768, and p53 in vivo. Together, they establish ZNF768 as an important protein controlling cell proliferation that could potentially be targeted to reduce tumorigenesis.

Recent studies indicate that ZNF768 depletion severely impairs proliferation in numerous cell lines [4, 5, 8]. As recently discussed, ZNF768 acts through independent but complementary mechanisms to regulate cell proliferation [7]. It was shown that ZNF768 controls the expression of a complex gene network to support cell cycle progression, mitosis, and cell division [4, 5]. We also found that ZNF768 physically interacts with p53 to repress its activity [4, 6]. The rapid degradation of ZNF768 in response to oncogenic and genotoxic stress was proposed as a checkpoint to decrease the expression of proliferative genes and to amplify p53 activation [7]. Supporting this model, we show herein that ZNF768 protein levels are rapidly reduced in mouse tissues following irradiation. Importantly, ZNF768 null mice showed a more important rise in the expression of several p53 targets following irradiation. In line with these results, we also found that primary cells isolated from ZNF768 null mice showed increased p53 levels and premature senescence following DNA damage and replicative stress. Together, these observations indicate that the interplay between ZNF768 and p53 occurs not only in cancer cell lines but also in primary cells and normal tissues.

Previous work indicates that high *ZNF768* mRNA expression is present in several human cancers including adrenocortical carcinoma, breast invasive carcinoma, cholangiocarcinoma, diffuse large B-cell lymphoma, kidney chromophobe carcinoma, kidney renal papillary cell carcinoma, low-grade glioma, liver hepatocellular carcinoma, pancreatic adenocarcinoma, LUAD, lung squamous cell carcinomas, and thymoma [4]. Tissue microarray analyses next confirmed that ZNF768 protein levels are extremely high in LUAD [8]. In this cancer, ZNF768 correlates with the mitotic score and Ki-67 expression, two classical markers of proliferation [8]. Here, high ZNF768 protein levels were observed in fibrosarcoma induced by the chemical carcinogen 3MC and in LUAD induced by oncogenic KRAS^G12D^. These observations provide further evidence that elevated ZNF768 is a common feature of proliferative cancer cells in vivo. Importantly, we show here that loss of ZNF768 significantly reduced lung tumor burden driven by KRAS^G12D^, confirming that ZNF768 plays a functional role in tumorigenesis. We also found a tendency for reduced tumor grade in mice depleted from ZNF768, supporting the idea that ZNF768 exerts pro-oncogenic functions, at least in LUAD.

It is interesting to point out that ZNF768 deletion was not sufficient to decrease fibrosarcoma development in mice injected with 3MC, and this despite a significant increase in intratumoral expression of the established p53 target CDKN1A/p21. As recently shown, tumors developing following 3MC exposure show a very distinctive and profound mutational landscape compared to tumors driven by oncogenic KRAS^G12D^ along with p53 loss [38]. In detail, 3MC-derived tumors exhibit nearly 80 times the median number of mutations compared to the oncogenic KRAS^G12D^ model, some of these mutations occurring in very important cancer driver genes. It is therefore possible that the loss of ZNF768 was not sufficient to repress proliferation in this highly mutagenic and aggressive cancer model. Additional studies are needed to define which cancer types are affected by ZNF768 loss and to precise the mechanisms involved in these effects.

Because acute ZNF768 depletion induces apoptosis and senescence and deeply impacts cell proliferation in cultured cells [4], we expected that ZNF768 loss would cause severe developmental defects in mice. Surprisingly, we only observed a mild growth defect in these animals. Although ZNF768 has unique structural features compared to other ZNFs, we cannot exclude the possibility that another member of this large family could have taken over to, at least partially, compensate for the loss of ZNF768. The high redundancy that exists within ZNFs makes this hypothesis highly plausible [39–41]. Although the basal phenotype of ZNF768 null mice was milder than expected, these mice showed elevated expression of p53 targets in various contexts, higher sensitivity to irradiation, and decreased tumor formation. Interestingly, these observations made in ZNF768 null mice are reminiscent of phenotypes previously reported in mouse models with overactive p53. For instance, mice haploinsufficient for *Mouse double minute 2* (*Mdm2*) or 4 (*Mdm4*) exhibit increased p53-dependent response to irradiation and delayed cancer development [32, 33, 42]. Consistently, mice carrying supernumerary copies of *p53* also exhibit enhanced *Cdkn1a/p21* expression in response to irradiation and protection against tumorigenesis [31]. In these studies, mice with elevated p53 were generally healthy and fertile and sometimes showed a minor decrease in body weight, as reported here. Overall, these observations further support the connection between ZNF768 and p53 in vivo.

In addition to exploring the link between ZNF768 and p53 in vivo, our study shows that ZNF768 loss affects the transcription of many genes outside of the p53 pathway. Previous studies have shown that ZNF768 is a transcription factor that binds to MIR sequences and regulates gene expression in a cell type-specific manner [5, 6]. Further analysis of the genes regulated by ZNF768 in multiple cell lines revealed that this transcription factor also controls the expression of a core set of genes that support proliferation including genes linked to cell cycle and mitosis [4]. Here, we found that loss of ZNF768 in mice reduced the expression of genes controlling different processes that participate in the integrated response to injury and stress, including transmembrane receptor signaling, cell adhesion, and growth [43–45]. Overall, our findings support a role for ZNF768 in regulating gene expression beyond the p53 network and give insights into the role of ZNF768 in modulating transcription upon genotoxic stress in vivo.

In conclusion, we show herein that ZNF768 levels positively associated with cell proliferation in vitro and in vivo. We propose that elevated ZNF768 expression may represent a novel mechanism to modulate p53 and gene expression to support tumorigenesis. Conversely, the rapid loss of ZNF768 following genotoxic stress could serve as a protective response to prevent proliferation of damaged cells. Defining the precise mechanisms regulating ZNF768 levels may offer novel opportunities to control cell proliferation and fight cancer.

## Supplementary information

Supplementary Figure legends

Figure S1

Figure S2

Figure S3

Figure S4

Figure S5

Table S1

## Data Availability

Abundance raw files presenting the levels of transcript for the RNA sequencing experiment can be found at this link: 10.5061/dryad.j0zpc86r3.
