# Supplementary material for: ZNF768 loss amplifies p53 action and reduces lung tumorigenesis in mice
Source: Oncogene. 2025 Mar 25;44(23):1793–804. doi: 10.1038/s41388-025-03352-w (PMC12143977; doi:10.1038/s41388-025-03352-w)
Supplement: Supplementary file 1 — Supplementary Figure legends [file 41388_2025_3352_MOESM1_ESM.docx]

**Figure S1. Generation of a ZNF7678 knockout mouse model.** (A) Genotyping analysis performed from tails of ZNF768 wild-type, heterozygous, and knockout mice. (B) Mendelian ratios calculated from the crossing of ZNF768 heterozygote mice. Significance was determined by Chi-square test. (C-D) Body weight and length of ZNF768 wild-type and knockout (C) male and (D) female mice (*n* = 5-9 animals/group) (21-22 weeks old). Significance was determined by 2-tailed, unpaired t test.

**Figure S2. ZNF768 loss does not hyperactivate p53 in mouse tissues in the basal state.** (A to C) qPCR analyses of gene involved in cell cycle, senescence, and cell death in the (A) thymus, (B) heart, and (C) liver of ZNF768 wild-type and knockout mice of both sexes (*n* = 5-9 animals/group). Data represent the mean ± SEM. Significance was determined by 2-tailed, unpaired t test. *P < 0.05 versus controls.

**Figure S3. *ZNF768* mRNA levels are not decreased following total body irradiation in mice.** (A-B) qPCR analysis of *Znf768* transcript levels in the heart of (A) male and (B) female wild-type mice 0, 2, 4, and 12 hours after 4 Gy total body irradiation (*n* = 3-4 animals/group). Data represent the mean ± SEM. Significance was determined by 1-way ANOVA. ***P < 0.01 versus controls.

**Figure S4. ZNF768 levels are induced in chemical and genetically-engineered cancer mouse models.** (A) Schematic representation of the strategy used to induce lung adenocarcinoma in LSL-*KRAS^G12D^; Trp53^fl/fl^* mice. The LSL-*KRAS^G12D^; Trp53^fl/fl^* mice carry the conditional activatable LSL-*KRAS*^G12D^ and the floxed *Trp53* allele. The LSL-*KRAS*^G12D^ allele comprises the *KRAS* gene carrying a point mutation (G12D) whose expression is blocked by the presence of a loxP-flanked stop codon. The floxed *Trp53* allele contains loxP sites flanking exons 2-10 of the *Trp53* gene. Following the intratracheal administration of Ad-Cre virus, the Cre-mediated recombination allows the expression of oncogenic *KRAS*^G12D^ and silences *Trp53* expression, leading to the development of lung tumors ranging from atypical adenomatous hyperplasia (AAH) to high grade adenocarcinoma. (B) ZNF768 immunohistochemistry and H&E staining on normal lung and lung tumors in LSL-*KRAS^G12D^; Trp53^fl/fl^* mice exposed to Ad-Cre. Low and high grade tumors are presented (magnification x 20, scale bars 50µm) (C) Quantification of ZNF768 immunochemistry staining using the H-score in lung and lung tumors of low and high grades (*n* = 9-10 tumors/group). Data are presented as box plots, where the center line represents the median, the box extends from the first to the third quartile and the whiskers indicate the minimum and maximum values. Significance was determined by 1-way ANOVA. *P < 0.05, ****P < 0.0001 versus controls.

**Figure S5. Loss of ZNF768 increases radiosensitivity and alters the transcriptional response to irradiation.** (A) ZNF768 wild-type and knockout MEFs were subjected to irradiation and were incubated for 14 days to allow colony formation. Colonies were fixed and stained with crystal violet and absorbance was measured following dissolution of the dye. Absorbance was reported on the non-irradiated control for both wild-type and knockout cell lines (*n* = 3 replicates/dose). Significance was determined by 2-way ANOVA. **P < 0.01 versus control. (B-C) Quantification of γ-H2AX in (B) thymus and (C) heart of wild-type and ZNF768 knockout of both sexes 0, 2, and 8 hours following 4 Gy total body irradiation. B-ACTIN was used as a loading control (*n* = 2-7 animals/group). Significance was determined by 2-way ANOVA. (D-F) qPCR analysis of p53 target genes in the (D) thymus of male and (E) heart and (F) thymus of female wild-type and ZNF768 knockout mice 2 and 8 hours after 4 Gy total body irradiation (*n* = 5-7 animals/group). Significance was determined by 2-way ANOVA. **P < 0.01 versus controls. (G) Heatmap showing changes in p53 target gene expression in the hearts of male wild-type and ZNF768 knockout mice 8 hours after 4 Gy total body irradiation (*n* = 6 animals/group). Significance was determined by Wald test with the Benjamini-Hochberg adjustment method. (H) Gene ontology analysis performed with Metascape on genes significantly downregulated (fold change <0.7, Padj < 0.01) in the heart of male ZNF768 null mice 8 hours after 4 Gy total body irradiation. In all panels, data represent the mean ± SEM.

**Supplementary Table 1. RNA sequencing analysis in the heart of wild-type and ZNF768 null male mice 8h after 4 Gy total body irradiation.**
