# Supplementary figures and images for: ZNF768 loss amplifies p53 action and reduces lung tumorigenesis in mice
Source: Oncogene. 2025 Mar 25;44(23):1793–804. doi: 10.1038/s41388-025-03352-w (PMC12143977; doi:10.1038/s41388-025-03352-w)

Figure S1. Generation of a ZNF768 knockout mouse model

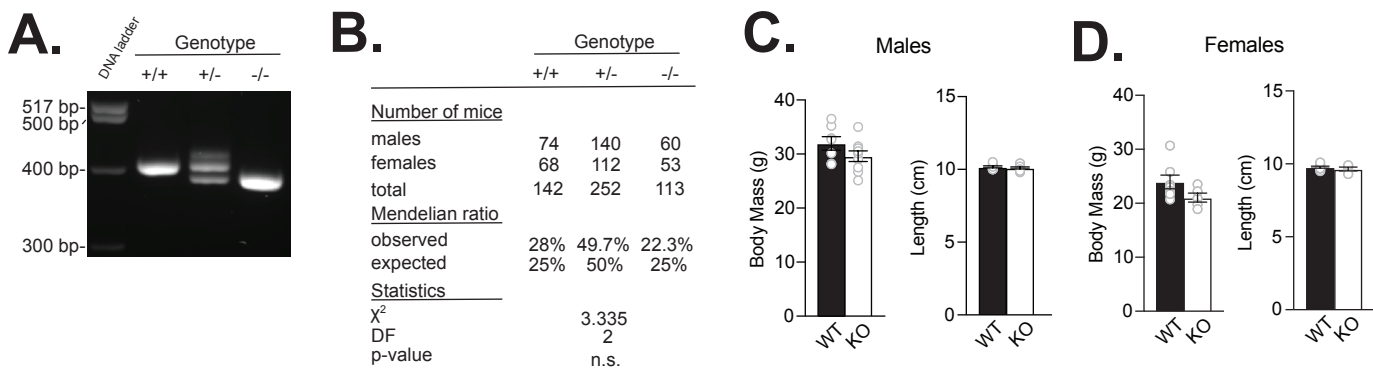

Supplement: Supplementary file 2 — Figure S1 [file 41388_2025_3352_MOESM2_ESM.pdf]

Figure S2. ZNF768 loss does not hyperactivate p53 in mouse tissues in basal state.

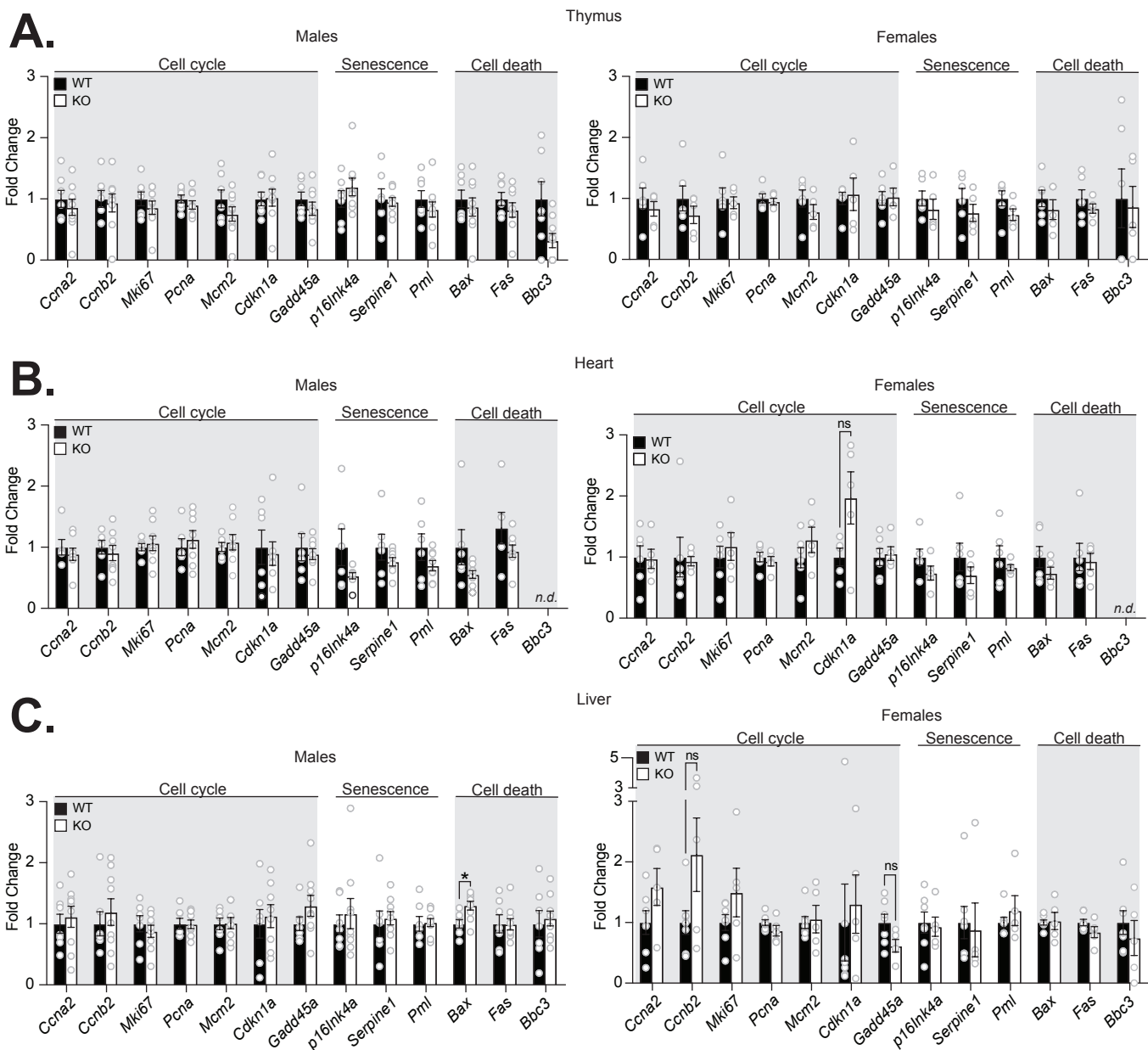

Supplement: Supplementary file 3 — Figure S2 [file 41388_2025_3352_MOESM3_ESM.pdf]

**Figure S3. ZNF768 mRNA levels are not decreased following total body irradiation in mice**

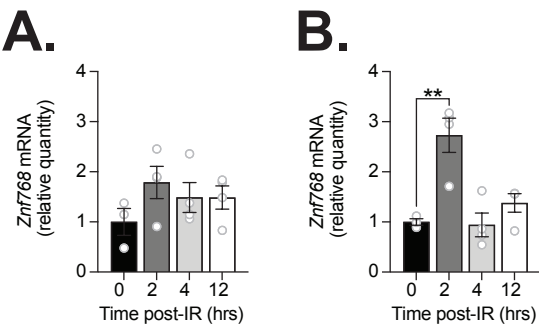

Supplement: Supplementary file 4 — Figure S3 [file 41388_2025_3352_MOESM4_ESM.pdf]

**Figure S4. ZNF768 levels are induced in chemical and genetically-engineered cancer mouse models**

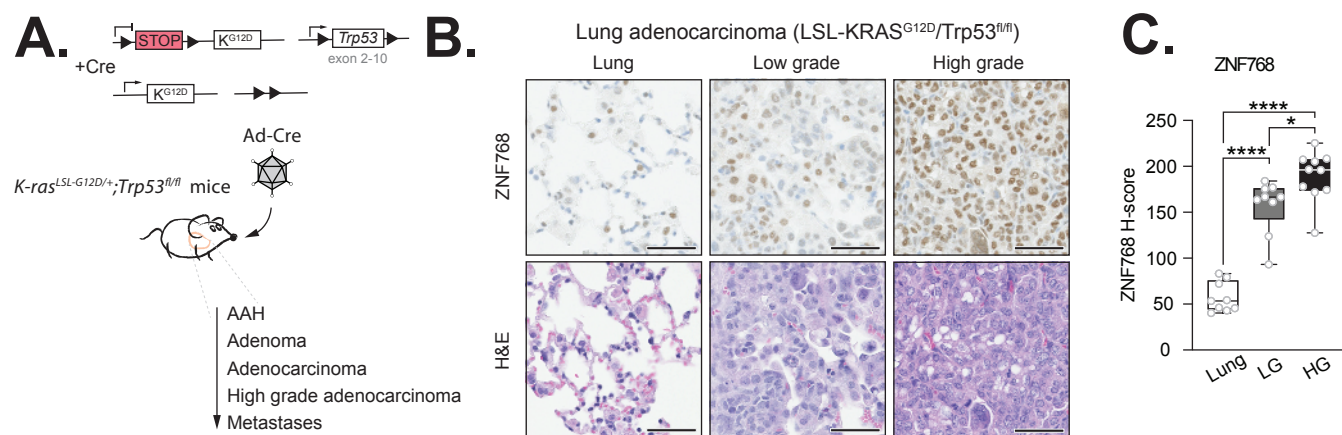

Supplement: Supplementary file 5 — Figure S4 [file 41388_2025_3352_MOESM5_ESM.pdf]
