# Supplementary material for: ZNF768 loss amplifies p53 action and reduces lung tumorigenesis in mice
Source: Oncogene. 2025 Mar 25;44(23):1793–804. doi: 10.1038/s41388-025-03352-w (PMC12143977; doi:10.1038/s41388-025-03352-w)

Figure S5. Loss of ZNF768 increases radiosensitivity and alters the transcriptional response to irradiation

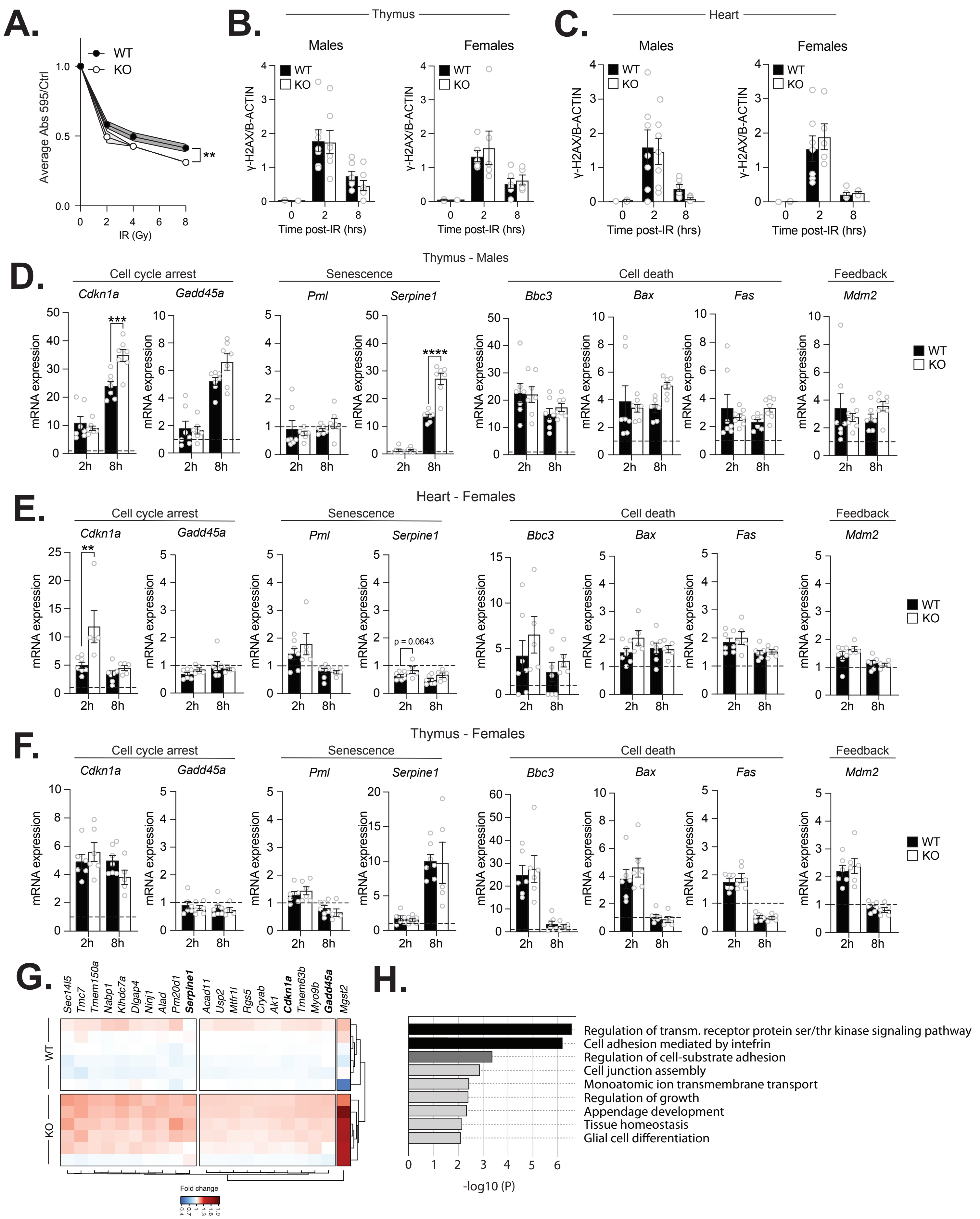

Supplement: Supplementary file 6 — Figure S5 [file 41388_2025_3352_MOESM6_ESM.pdf]
